# Socioeconomics, health-related factors, and tooth loss among the population aged over 80 years in China

**DOI:** 10.1186/s12889-022-12861-2

**Published:** 2022-03-05

**Authors:** Hanmo Yang, Runlin Han, Zhenjie Wang

**Affiliations:** 1 National School of Development, Peking University, Beijing, 100871 People’s Republic of China; 2 Institute of World Economics and Politics, Chinese Academy of Social Sciences, Beijing, 100732 People’s Republic of China; 3 Institute of Population Research, Peking University, Beijing, 100871 People’s Republic of China

**Keywords:** Tooth loss, Odds ratio, Oldest-old, Dietary intake

## Abstract

**Background:**

The prevalence of tooth loss varies across the globe among oldest-old individuals. The presence of fewer than 20 teeth in old age was associated with a decrease in people’s health and quality of life. This paper explored the association between socioeconomics, health-related factors, and tooth loss among the population over the age of 80 in China.

**Methods:**

The tooth loss status of older Chinese adults was collected with a structured questionnaire from the 8^th^ wave of the Chinese Longitudinal Healthy Longevity Survey (CLHLS). A total of 6716 individuals aged 80 years and above were included. Logistic regression was used to assess the association between socioeconomic statuses, dietary intake at approximately 60 years old, health-related factors, and tooth loss.

**Results:**

Of the 6716 individuals aged 80 years and above, the composition of the group with fewer teeth for both men and women was statistically significant in many ways. Multivariate logistic regression analyses show that for men, being older than 90 years and being ADL disabled (adjusted OR: 1.71, 95% CI: 1.01–2.89) are factors that are significantly and consistently associated with a higher risk of having fewer than 20 teeth, while having a higher household income per capita (adjusted OR: 0.56, 95% CI: 0.32–0.99) decreases the risk. For women, an age of above 95 years, brushing teeth less than once per day (adjusted OR: 1.96, 95% CI: 1.26–3.03), consuming sugar some of the time as opposed to less than once per month at approximately 60 years old (adjusted OR = 1.74, 95% CI: 1.15–2.62), and being ADL disabled (adjusted OR: 1.70, 95% CI: 1.04–2.77) are factors that are significantly associated with the risk of having fewer than 20 teeth.

**Conclusion:**

The analysis suggests that socioeconomic status, dietary intake in early old age, and ADL capacity are associated with the risk of having fewer teeth for the population aged 80 years and above, and the risk factors vary between sexes.

## Background

Dental and oral health is essential to maintaining eating and communication abilities and is thus a significant factor in people’s overall health and quality of life [1]. As a key indicator of oral health, tooth loss is widely prevalent in China and across the globe, especially among the population aged 80 years and above [2, 3]. According to the Global Burden of Diseases, Injuries, and Risk Factors Study 2017 (GBD 2017), oral disorders, such as caries, periodontal disease, edentulism, and severe tooth loss, were the top-ranked most common causes of prevalence of and years lived with disability (YLD) for both sexes separately in 1990, 2007 and 2017 [2]. In 2017, more than 2.67 billion people suffered from severe tooth loss and edentulism around the world, an increase of 40.8% since 1990 [2, 3]. In China, the 4^th^ National Oral Health Survey suggests that 5.8% of the population between 65 and 74 years old was edentulous, and the proportion of people with more than 20 remaining teeth was 73.6%, which is significantly higher in urban than in rural areas [4].

The WHO proposed an “8020” campaign to encourage individuals to retain 20 or more teeth at the age of 80 because having at least 20 teeth is required to preserve a basic masticatory function [5], and many research efforts suggest that people with 20 or more teeth have a higher level of health and healthy life expectancy [6–9]. Some studies have suggested that cigarette smoking [10–13], sugar consumption [14, 15], alcohol consumption [12, 16], and poor oral health habits, such as a low frequency of toothbrushing, are risk factors for tooth loss [17, 18]. The consumption of green tea [19, 20], mushrooms [21, 22], milk [23], and vitamin D intake were associated with a decreased risk of tooth loss [24]. Moreover, some studies have suggested that overweight (BMI ≥ 25) or obese people (BMI ≥ 30) or people with hypertension or diabetes may have a higher risk of losing teeth in middle- and high-income countries [25–28]. Moreover, there is evidence suggesting a positive correlation between socioeconomic status and tooth loss in European and East Asian countries [29–32].

Very few studies have investigated the association between socioeconomic status, dietary intake at approximately 60 years old, chronic diseases and tooth loss among Chinese men and women in the recent decade. This study explores the association between socioeconomics, health-related factors, and tooth loss among the population aged over 80 years in China.

## Methods

### Study design and participants

We draw data from the 8^th^ wave of the Chinese Longitudinal Healthy Longevity Survey (CLHLS) conducted in 2017–2019 and released in 2020 [33]. To avoid the problem of small subsample sizes at more advanced ages, the sampling design in the first wave follows a multistage disproportionate and targeted random sampling procedure: the CLHLS included face-to-face interviews with all centenarians (people aged 100 or above) living in a randomly selected half of the counties and cities in 22 of 31 Chinese provinces, covering approximately 85 percent of the total population in China. For each centenarian selected, two residents living nearby (same village or street) were also interviewed. Residents living nearby were randomly selected for age and sex according to the survey ID code of centenarians; only one of them was 80–89 years old, while the other was 90–99 years old [34]. To adjust the oversampled population of more advanced ages, CHLHS provides age-sex- and rural-urban-specific weights. The method for computing the weight and related discussion can be found elsewhere [35].

The interview was conducted at the interviewee’s home with some basic physical capacity tests performed. The survey covered questions regarding various aspects of the lives of the participants, such as demographic information, family structure, dietary intake, chronic disease, medical care, cigarette smoking and alcohol consumption, nutrition and health-related conditions in early life. The interview refusal rate among the Chinese oldest-old was very low because they do in general like to talk to other people, and many of them are proud to be recognized as members of the long-lived group. For disabled people, many agree to participate through proxy assistance by a close family member [35].

To maintain a sufficiently large sample size, the CLHLS replaced deceased participants with new respondents in each follow-up wave. In the 8^th^ wave, the questionnaires were completed by 10,427 individuals aged 80 years or above (4175 men, 6252 women), among which 3711 were excluded due to incomplete data, yielding 6716 individuals (2676 men and 4040 women) as the subjects of our analyses.

The design of CLHLS was approved by the Campus Institutional Review Board of Duke University (Pro00062871) and the Biomedical Ethics Committee of Peking University (IRB00001052-13,074). The research was performed in accordance with the Declaration of Helsinki. Informed consent was obtained from all participants during the face-to-face interview.

### Assessment of tooth loss

The survey collected information on the number of teeth by asking, “How many natural teeth does the interviewee have?” (excluding false teeth). We grouped the participants as having fewer than 20 teeth and having 20 or more teeth. We chose 20 natural teeth as the threshold according to the WHO “8020” campaign [5]. Extensive studies have suggested that having 20 or more teeth is associated with a higher level of health and quality of living [6–9]. Nevertheless, this survey was not specifically designed to study dental health; thus, no questions about dental caries, periodontal diseases, or dental checkups were asked.

### Assessment of dietary intake and health behaviors

The subjects were asked about the frequencies of dietary intake of more than twenty kinds of foods and drinks at approximately 60 years of age and at present, which were categorized into five groups: “rarely or never”, “not every month but occasionally”, “not every week but at least once per month”, “not every day but at least once per week”, and “almost every day”. In this study, we merged the first two answers into 0 (less than once per month), merged the third and fourth answers into 1 (some of the time), and recoded Group 3 (almost every day). We selected the consumption of fish, mushrooms, milk, green tea, and sugar as the nutritional factors of interest. To avoid the problem of reciprocal causation, we used the frequency of consumption at approximately age 60 instead of current consumption to explore its impact on the status of tooth loss. Due to data limitations, we were unable to include the amount of dietary intake in our research.

Cigarette smoking and alcohol consumption habits in the past or at present were considered two nonnegligible factors. The survey collected information on cigarette smoking and alcohol consumption with the following questions: “Do you smoke at the present time?”, “Did you smoke in the past?”, “Do you drink alcohol at the present time?”, and “Did you drink alcohol in the past?”, which allowed us to identify former and current smokers or drinkers from those who never drink alcohol or smoke cigarettes. For the 3723 individuals who had at least one tooth, we further identified their oral hygiene habits from the question “How often do you brush your teeth?”. The questionnaire measures the frequency of toothbrushing using six categories: 1 (do not), 2 (sporadically), 3 (once per day), 4 (twice per day), 5 (thrice or more per day), and 6 (unknown), and we recategorized the answers into 1 (less than once per day) and 0 (once or more per day).

### Assessment of other covariates

We used overweight, activities of daily living (ADL), and chronic diseases as measures of health. We calculated the respondents’ BMIs based on their weight and height measured by the interviewers and identified those who were overweight (BMI ≥ 25) [36]. Self-reported hypertension and diabetes were categorized into 1 (yes) and 0 (no). There are six indicators assessing the respondents’ ADL capacity in CLHLS using the Katz ADL index: eating, dressing, indoor transferring, toileting, continence, and bathing [37]. If a participant needed help to perform daily functions in at least one of the six activities, then the person was coded 1 (disabled in ADL) as opposed to 0 (not disabled in ADL).

To evaluate the socioeconomic statuses of the interviewees, we used current residence area (1 = “rural”, 0 = “urban”), marital status (1 = “unmarried (widowed/divorced/never married)”, 0 = “married”), age, household income, and educational status as indicators. Age was categorized into five groups (0 = “80–84”, 1 = “85–89”, 2 = “90–94”, 3 = “95–99”, and 4 = “ ≥ 100”), and household income was divided into five 20-percentile groups (0 = “0-20^th^ percentile”, 1 = “21^st^-40^th^ percentile”, 2 = “41^st^-60^th^ percentile”, 3 = “61^st^-80^th^ percentile”, and 4 = “81^st^-100^th^ percentile”). Among the 6716 subjects, 34.57% of men and 79.85% of women had never entered school; thus, we used a dummy variable to differentiate people who were “illiterate” or “had at least one year of education”, coded 1 and 0, respectively.

### Statistical analysis

We compared individuals with fewer than 20 teeth and with 20 or more teeth by sex, illustrated the distribution of characteristics using one-way analysis of variance, and conducted the chi-square test. Since the interviewees of more advanced ages were oversampled, appropriate weights were employed to ensure that the results were nationally representative and internationally comparable. We weighted the samples based on the age-sex- and rural-urban-specific distribution of the 2015 minicensus. Univariate logistic regression analysis was conducted to calculate the crude odds ratios (ORs) and their 95% confidence intervals (95% CIs) for having fewer than 20 teeth. Multivariate logistic regression analysis was also conducted to estimate the adjusted odds ratios at 95% confidence intervals with regard to all variables, since the crude ORs are either statistically significant or the variables are of academic interest. Statistical significance was set at *p* < 0.05. Statistical analyses were performed using Stata 16.

## Results

Among our weighted samples, 56.61% were females, and the weighted mean ages of male and female subjects were 84.19 and 84.48 years old, respectively. The proportion of people with fewer than 20 teeth was higher among women (81.36%) than among men (74.70%). Table 1 shows the characteristic distribution of males and females with fewer than 20 teeth and those with more than or 20 teeth. The composition of the group with fewer teeth was different from that of the other group at a statistically significant level in terms of age, household income per capita, current residence area, marital status, educational level, and whether teeth were brushed every day for men and in all aspects except for the current residence area for women. For dietary intake at approximately 60 years of age and health conditions, the levels of significance of the risk factors were different between sexes. The proportion of people who drink alcohol at present is 9.03% for males and 1.84% for females. No statistically significant differences in alcohol consumption habits were found between the group with fewer and more teeth. The proportion of current smokers among male subjects was 23.19%, and 5.02% among female subjects. Men who had never smoked were more likely to have more teeth, and those who were previous or current smokers were more likely to have fewer teeth.

**Table 1:** Characteristics of subjects (%)

		Males			Females		
		Number of teeth			Number of teeth		
		< 20	≥ 20	*p* value	< 20	≥ 20	*p* value
		%	%		%	%	
Number of participants (weighted)		1787	605		2539	582	
Age	80–84	59.04	69.80		55.58	70.16	
	85–89	27.19	24.31		30.71	23.90	
	90–94	11.35	5.21		10.71	5.16	
	95–99	2.22	0.64		2.65	0.73	
	≥100	0.21	0.04	< 0.001	0.36	0.06	< 0.001
Household income per capita	Q1	23.58	12.55		27.49	22.67	
	Q2	18.76	12.97		18.61	13.84	
	Q3	17.76	18.96		20.19	19.69	
	Q4	21.09	26.56		17.73	19.63	
	Q5	18.81	28.96	< 0.001	15.98	24.17	0.023
Current residence area	Urban	53.38	68.26		53.18	58.39	
	Rural	46.62	31.74	< 0.001	46.82	41.61	0.163
Marital status	Married	59.01	66.87		26.80	34.16	
	Unmarried	40.99	33.13	0.020	73.20	65.84	0.032
Illiterate	No	69.71	78.55		27.34	38.80	
	Yes	30.29	21.45	0.005	72.66	61.20	< 0.001
Brushing less than once per day^a^	No	69.71	78.55		67.45	82.50	
	Yes	30.29	21.45	< 0.001	32.55	17.50	< 0.001
Sugar consumption	Less than once per month	59.03	57.78		60.81	69.29	
	Some of the time	34.11	30.12		30.30	22.47	
	Almost everyday	6.86	12.10	0.020	8.89	8.24	0.043
Green tea consumption	Less than once per month	85.65	79.28		93.12	89.69	
	Some of the time	2.83	2.14		1.60	2.63	
	Almost everyday	11.52	18.58	0.012	5.28	7.68	0.228
Mushroom consumption	Less than once per month	67.82	57.96		66.97	62.00	
	Some of the time	30.14	39.20		30.62	34.82	
	Almost everyday	2.04	2.83	0.009	2.41	3.17	0.339
Fish consumption	Less than once per month	39.17	26.62		43.42	37.30	
	Some of the time	53.27	62.08		47.77	54.58	
	Almost everyday	7.55	11.30	< 0.001	8.82	8.12	0.163
Milk consumption	Less than once per month	68.90	53.32		66.41	60.59	
	Some of the time	19.17	24.83		20.96	21.26	
	Almost everyday	11.94	21.85	< 0.001	12.64	18.15	0.076
Alcohol consumption	Never	79.81	80.92		97.33	96.90	
	Drank in the past	9.93	8.97		1.06	1.75	
	Drink at present	10.25	10.11	0.892	1.61	1.35	0.648
Cigarette smoking	Never	41.12	49.96		88.90	90.96	
	Previous smoker	30.47	32.12		5.53	3.80	
	Current smoker	28.41	17.93	0.002	5.58	5.24	0.557
ADL capacity	Not disabled	84.03	90.82		81.81	89.68	
	Disabled	15.97	9.18	0.004	18.19	10.32	0.002
Hypertension	No	58.37	50.65		52.15	48.14	
	Yes	41.63	49.35	0.029	47.85	51.86	0.279
Diabetes	No	89.59	83.74		88.35	83.24	
	Yes	10.41	16.26	0.012	11.65	16.76	0.035
Overweight (BMI ≥ 25)	No	78.96	72.16		75.57	71.26	
	Yes	21.04	27.84	0.031	24.43	28.74	0.199

^a^2993 with no teeth were excluded

The crude odds ratios of the variables associated with having fewer than 20 teeth are shown in Table 2. Being older, unmarried, illiterate, and brushing teeth less than once per day are independently and significantly associated with having fewer than 20 teeth for both men and women. Other risk factors for having fewer teeth, including current residence area, dietary intake at approximately 60 years old, and health-related factors, show different patterns for men and women.

**Table 2:** Crude odds ratios and 95% confidence intervals of the variables associated with having < 20 teeth

		Males				Females				
Variable		Number of teeth (< 20)				Number of teeth (< 20)				
		Crude OR	95% CI		*P* value	Crude OR	95% CI		*P* value	
Age	80–84	Reference				Reference				
	85–89	1.323	0.981	1.783	0.066	1.622	1.177	2.236		0.003
	90–94	2.574	1.901	3.485	< 0.001	2.619	1.866	3.676		< 0.001
	95–99	4.119	2.664	6.367	< 0.001	4.602	2.852	7.426		< 0.001
	≥100	6.013	3.570	10.13	< 0.001	8.066	5.278	12.32		< 0.001
Household income per capita	Q1	Reference				Reference				
	Q2	0.770	0.461	1.287	0.319	1.110	0.687	1.794		0.671
	Q3	0.499	0.306	0.812	0.005	0.846	0.540	1.325		0.464
	Q4	0.423	0.268	0.667	< 0.001	0.745	0.475	1.167		0.198
	Q5	0.346	0.220	0.542	< 0.001	0.545	0.355	0.839		0.006
Current residence area	Rural (Ref. Urban)	1.878	1.386	2.545	< 0.001	1.236	0.918	1.663		0.163
Marital status	Unmarried (Ref. Married)	1.402	1.053	1.866	0.021	1.417	1.029	1.950		0.033
Illiterate	Yes (Ref. No)	1.591	1.149	2.203	0.005	1.685	1.244	2.283		< 0.001
Brushing less than once per day^a^	Yes (Ref. No)	2.347	1.656	3.324	< 0.001	2.276	1.538	3.367		< 0.001
Green tea consumption	Less than once per month	Reference				Reference				
	Some of the time	1.222	0.500	2.984	0.660	0.586	0.211	1.63		0.306
	Almost everyday	0.574	0.388	0.849	0.005	0.661	0.376	1.164		0.152
Mushroom consumption	Less than once per month	Reference				Reference				
	Some of the time	0.657	0.491	0.880	0.005	0.814	0.598	1.109		0.192
	Almost everyday	0.614	0.276	1.368	0.233	0.704	0.303	1.635		0.414
Fish consumption	Less than once per month	Reference				Reference				
	Some of the time	0.583	0.427	0.796	0.001	0.752	0.552	1.024		0.070
	Almost everyday	0.454	0.273	0.756	0.002	0.933	0.550	1.583		0.796
Milk consumption	Less than once per month	Reference				Reference				
	Some of the time	0.598	0.422	0.846	0.004	0.899	0.626	1.292		0.566
	Almost everyday	0.423	0.294	0.609	< 0.001	0.635	0.428	0.942		0.024
Sugar consumption	Less than once per month	Reference				Reference				
	Some of the time	1.108	0.812	1.512	0.516	1.537	1.088	2.171		0.015
	Almost everyday	0.555	0.348	0.883	0.013	1.230	0.734	2.061		0.432
Alcohol consumption	Never drink	Reference				Reference				
	Drank in the past	1.123	0.699	1.805	0.630	0.604	0.213	1.71		0.342
	Drink at present	1.028	0.641	1.648	0.909	1.182	0.322	4.339		0.801
Cigarette smoking	Never smoked	Reference				Reference				
	Previous smoker	1.153	0.837	1.587	0.383	1.488	0.716	3.094		0.287
	Current smoker	1.925	1.324	2.800	< 0.001	1.089	0.566	2.093		0.799
ADL capacity	Disabled (Ref. Not disabled)	1.881	1.219	2.903	0.004	1.933	1.278	2.924		0.002
Hypertension	Yes (Ref. No)	0.712	0.531	0.955	0.024	0.850	0.628	1.151		0.293
Diabetes	Yes (Ref. No)	0.598	0.400	0.896	0.013	0.655	0.441	0.973		0.036
Overweight (BMI ≥ 25)	Yes (Ref. No)	0.690	0.493	0.968	0.031	0.802	0.572	1.123		0.199

^a^2993 with no teeth were excluded

Table 3 shows the results of multivariate unconditional logistic regressions for men and women. For males, being in an age group of 90 years and above meant they were 1.92 (95% CI: 1.32–2.79) to 2.78 (95% CI: 1.35–5.71) times more likely to have fewer than 20 teeth. Males with a higher household income per capita had a lower chance of having fewer teeth, with adjusted ORs of 0.56 (95% CI: 0.315–0.986) for the top quintile. For females, those aged 95–99 years old and centenarians had a 2.02- to 3.09-fold greater chance of having fewer than 20 teeth (95% CI: 1.08–3.77, 95% CI: 1.72–5.55, respectively), while women who brushed their teeth less than once per day had a 1.96-fold greater chance of having fewer teeth (95% CI: 1.26–3.03). Consuming sugar some of the time as opposed to less than once per month (adjusted OR: 1.74, 95% CI: 1.15–2.62) for women and being ADL disabled for men (adjusted OR: 1.71, 95% CI: 1.01–2.89) and women (adjusted OR: 1.70, 95% CI: 1.04–2.77) were significantly associated with the risk of having fewer than 20 teeth.

**Table 3:** Adjusted odds ratios and 95% confidence intervals of the variables associated with having < 20 teeth^a^

		Males				Females			
Variable		Number of teeth (< 20)				Number of teeth (< 20)			
		Adjusted OR	95%CI		*p* value	Adjusted OR	95%CI		*p* value
Age	80–84	Reference				Reference			
	85–89	1.190	0.821	1.724	0.358	1.199	0.824	1.746	0.342
	90–94	1.923	1.323	2.794	< 0.001	1.383	0.894	2.14	0.145
	95–99	2.739	1.539	4.876	< 0.001	2.022	1.084	3.773	0.027
	≥100	2.780	1.354	5.709	0.005	3.088	1.718	5.549	< 0.001
Household income per capita	Q1	Reference				Reference			
	Q2	0.930	0.496	1.741	0.820	1.193	0.675	2.106	0.543
	Q3	0.656	0.37	1.161	0.148	0.814	0.485	1.363	0.433
	Q4	0.602	0.339	1.069	0.083	0.664	0.381	1.157	0.148
	Q5	0.557	0.315	0.986	0.045	0.691	0.41	1.164	0.165
Current residence area	Rural (Ref. Urban)	1.418	0.961	2.093	0.079	0.985	0.665	1.46	0.940
Marital status	Unmarried (Ref. Married)	1.061	0.750	1.500	0.738	1.209	0.823	1.777	0.334
Illiterate	Yes (Ref. No)	1.156	0.772	1.732	0.480	0.944	0.645	1.382	0.767
Brushing less than once per day	Yes (Ref. No)	1.346	0.887	2.043	0.163	1.957	1.263	3.034	0.003
Green tea consumption	Less than once per month	Reference				Reference			
	Some of the time	1.500	0.52	4.328	0.453	0.725	0.231	2.278	0.582
	Almost everyday	0.804	0.495	1.304	0.376	1.225	0.638	2.352	0.541
Mushroom consumption	Less than once per month	Reference				Reference			
	Some of the time	1.062	0.722	1.561	0.760	1.143	0.762	1.715	0.519
	Almost everyday	1.285	0.48	3.439	0.617	0.951	0.316	2.86	0.929
Fish consumption	Less than once per month	Reference				Reference			
	Some of the time	0.970	0.651	1.446	0.883	0.939	0.637	1.383	0.749
	Almost everyday	0.795	0.414	1.525	0.489	1.572	0.818	3.02	0.174
Milk consumption	Less than once per month	Reference				Reference			
	Some of the time	0.697	0.452	1.075	0.102	0.970	0.632	1.489	0.889
	Almost everyday	0.733	0.442	1.216	0.229	0.683	0.411	1.137	0.142
Sugar consumption	Less than once per month	Reference				Reference			
	Some of the time	1.245	0.853	1.816	0.256	1.739	1.154	2.619	0.008
	Almost everyday	0.627	0.353	1.114	0.111	0.947	0.528	1.7	0.855
Alcohol consumption	Never drink	Reference				Reference			
	Drank in the past	1.166	0.645	2.109	0.611	0.540	0.17	1.713	0.295
	Drink at present	0.901	0.51	1.593	0.720	1.356	0.291	6.323	0.698
Cigarette smoking	Never smoked	Reference				Reference			
	Former smoker	1.116	0.753	1.656	0.584	1.609	0.695	3.726	0.267
	Current smoker	1.504	0.948	2.387	0.083	0.779	0.356	1.707	0.533
ADL capacity	Disabled (Ref. Not disabled)	1.710	1.011	2.891	0.045	1.696	1.039	2.768	0.035
Hypertension	Yes (Ref. No)	0.936	0.66	1.327	0.710	1.066	0.745	1.526	0.726
Diabetes	Yes (Ref. No)	1.015	0.61	1.691	0.953	0.720	0.436	1.19	0.200
Overweight (BMI ≥ 25)	Yes (Ref. No)	0.820	0.55	1.225	0.083	0.734	0.497	1.085	0.121

^a^2993 with no teeth were excluded

## Discussion

In the current study, older age and ADL disability are significantly associated with fewer natural teeth in males, while having higher household income per capita decreases the risk. For women, having an age of 95 years and above, brushing teeth less than once per day, consuming sugar some of the time as opposed to less than once per month, and being ADL disabled are associated with having fewer natural teeth.

Consistent with many previous studies in various populations, aging is a major risk factor for tooth loss [18, 30, 38]. Our analysis results also suggest a significant association between lower household income and fewer teeth for males. As a key indicator of socioeconomic status, household income was found to be correlated with the number of remaining teeth in many other studies [29, 39]. Other socioeconomic risk factors, namely, current residence area, marital status, and educational level, are significantly associated with the risk of having fewer than 20 teeth independently but not in the multivariate logistic regression. Our results confirmed the results of other studies suggesting that males who lived in rural areas were more likely to have fewer teeth than their urban counterparts (crude OR = 1.88, 95% CI: 1.39–2.55). A study found a similar result for 3767 Korean adults aged 55–84, although it did not distinguish sex differences, and argued that compared to urban areas, a lower standard of living and less availability, accessibility, and quality of dental services in rural areas may result in lack of prevention and treatment of oral diseases [40]. Regarding marital status, our analyses found that those who were currently not married were more likely to have fewer teeth than those who were married (crude OR = 1.40, 95% CI: 1.05–1.87 for men; crude OR = 1.42, 95% CI: 1.03–1.95 for women). This echoes the findings of a longitudinal study among 3033 Canadians over 50 years of age, which indicated that the probability of losing one or more teeth among people who are not currently married is significantly higher [41]. An intuitively similar association exists between tooth loss and living alone, which was found only among women in another study based in Italy [30]. As an important indicator of socioeconomic status, educational attainment has been proven to be associated with the number of teeth [29, 38]. In the current study, being illiterate was independently associated with having fewer than 20 teeth for both men (crude OR = 1.60, 95% CI: 1.15–2.20) and women (crude OR = 1.69, 95% CI: 1.24–2.28). Nevertheless, the multivariate logistic analysis does not give a consistent result on those socioeconomic risk factors once we took household income per capita into consideration.

Women are more vulnerable to risk factors in terms of living habits and dietary intake in early old age. Tooth loss and dental caries can be attributed to dental plaque, and regular tooth brushing is an effective way to remove dental plaque and improve oral hygiene [42, 43]. Our multivariate logistic regression results suggest that brushing teeth less than once per day nearly doubles the risk of having fewer teeth for women (adjusted OR = 1.96, 95% CI: 1.26–3.03), while the association among men is less significant. In addition, our results show that consuming sugar more frequently is associated with a higher risk of having fewer than 20 teeth for women (adjusted OR = 1.74, 95% CI: 1.15–2.62). The associations between fewer teeth and the frequency of consumption of sugar are consistent with previous research in general [44, 45]. The reason behind this result is that consumed sugar interacts with bacteria within dental plaque and causes acid, which attacks the enamel and dentine of teeth. More frequent sugar consumption keeps the pH of the plaque below a critical level for demineralization, causing caries and eventually leading to tooth loss [46]. Indeed, poor oral hygiene and lack of awareness are the top causes of poor dental status in China. According to an epidemiological survey of 6843 individuals in medium and small cities and towns across China, only 8.9% of the interviewees had regular dental checkups in 2009 [3]. Our findings highlight the importance of oral hygiene and oral health habits by providing sex-specific evidence for the oldest-old.

Many research efforts have suggested that cigarette smoking is a significant risk factor for tooth loss. In the current study, the independent association between cigarette smoking and tooth loss in the crude OR analysis was statistically significant for men (crude OR: 1.93, 95% CI: 1.32–2.80), but the explanatory power of this correlation diminished when we added more risk factors to the logistic function. Some scholars argued that cigarette smoking acts as a link between socioeconomic status and tooth loss through education or income [29]. For example, people with lower education are more often smokers than their peers with higher education. This is not the case in our sample, where 80.71% of illiterate people had never smoked, and the proportion was only 59.08% in the group who had at least one year of education. Others have suggested that cigarette smoking affects the number of teeth through periodontal diseases; thus, it cannot be regarded as an independent risk factor for fewer teeth [47].

Previous studies have provided inconsistent evidence of the impact of alcohol consumption on various populations. Some results suggested a negative impact of alcohol consumption on dental health [16, 48]. In contrast, a study of 8352 men aged 40 to 79 years in Japan showed that current drinkers had a significantly lower risk of having fewer than 20 teeth [49]. Another study on a Japanese population aged 60 years or above based on analyses of the national database also concluded that the crude and adjusted ORs of former and current drinkers were both < 1 [12], and the authors explained that it was possibly due to the much less pronounced mucosal irritation caused by Japanese sake than by ethanol or whiskey [50]. In the current study, regardless of whether we limited the alcohol category to “very strong liquor (≥ 38%)” or alcohol in general, the results were not significant in either sex.

Although some previous studies found positive associations between certain chronic diseases and dental disorders [27, 28], our study reveals no such results. One reason is that our study population is in the oldest-old age group; thus, the correlation between chronic diseases and dental health could be different from what has been found among the younger population in other studies [51]. Another study based on the 8^th^ wave of CLHLS data found that prevalence rates of hypertension and diabetes decreased significantly with increasing age (*P* < 0.05) [52]. In that case, the crude ORs of hypertension and diabetes in our study partly reveal the association between age and the risk of tooth loss. As we controlled for age and other risk factors in the logistic functions, such correlations were no longer statistically significant.

Our study has several strengths, including the large sample size, the population-based design, and adjustment for a wide range of socioeconomic characteristics and health-related factors. However, our study also has several limitations, which should be taken into consideration by future researchers. As a cross-sectional study, we cannot deduce causative pathways between tooth loss and chronic diseases; therefore, future longitudinal research is needed to assess the causal relationships between tooth loss, chronic diseases, and socioeconomic factors. Moreover, residual confounding by other unmeasured or unknown factors remains possible. We cannot investigate the type of time of tooth loss and denture use, dental symptoms, and dental care/utilization, as these data were not collected. Our study included a sample of very old age. The participants were older than the general aged population in China. The main study findings, including the evaluation of the optimal number of natural teeth, may not be applicable to other populations.

## Conclusion

Using data from the 8th wave Chinese Longitudinal Healthy Longevity Survey (CLHLS), we explored the risk factors for tooth loss among the population over the age of 80 in China. Tooth loss is widely prevalent across the globe among the oldest-old. The presence of fewer than 20 teeth in old age is associated with a decrease in people’s health and quality of life. The results suggested that having an older age and being ADL disabled are significantly associated with few natural teeth for both sexes, while having a higher household income per capita decreases the risk for males. For females, brushing less than once per day and some dietary habits in early old age are significantly associated with the risk of having fewer than 20 teeth. The results underline the importance of preventing tooth loss among the oldest-old population to improve the quality of life in this rapidly growing population.

## Data Availability

This study was based on datasets from the Chinese Longitudinal Healthy Longevity Survey (CLHLS) conducted in longevity areas. The CLHLS data can be publicly obtained through Peking University Open Research Data (https://opendata.pku.edu.cn/dataverse/CHADS).

## Abbreviations

ADL: Activities of daily living
BMI: Body mass index
CI: Confidence interval
CLHLS: Chinese Longitudinal Healthy Longevity Survey
OR: Odds ratio
WHO: World Health Organization
YLD: Years lived with disability

## References

[1] Kandelman D, Petersen PE, Ueda H (2008). Oral health, general health, and quality of life in older people. Spec Care Dentist.

[2] James SL, Abate D, Abate KH, Abay SM, Abbafati C, Abbasi N (2018). Global, regional, and national incidence, prevalence, and years lived with disability for 354 diseases and injuries for 195 countries and territories, 1990–2017: a systematic analysis for the Global Burden of Disease Study 2017. Lancet.

[3] Rong W. A national survey on dentin hypersensitivity in Chinese adults in rural areas. In: Report on Chinese Oral Health (2012). Beijing: Social sciences academic press; 2012.

[4] Guo J, Ban JH, Li G, Wang X, Feng XP, Tai BJ (2018). Status of tooth loss and denture restoration in Chinese adult population: findings from the 4th National Oral Health Survey. Chin J Dent Res.

[5] WHO. Recent Advances in Oral Health. WHO Technical Report Series No. 826. Geneva: World Health Organization; 1992. 1462607

[6] Matsuyama Y, Aida J, Watt RG, Tsuboya T, Koyama S, Sato Y (2017). Dental status and compression of life expectancy with disability. J Dent Res.

[7] Yamamoto T, Kondo K, Hirai H, Nakade M, Aida J, Hirata Y (2012). Association between self-reported dental health status and onset of dementia: a 4-year prospective cohort study of older Japanese adults from the Aichi Gerontological Evaluation Study (AGES) Project. Psychosom Med.

[8] Aida J, Kondo K, Hirai H, Nakade M, Yamamoto T, Hanibuchi T (2012). Association between dental status and incident disability in an older Japanese population. J Am Geriatr Soc.

[9] Yamanaka K, Nakagaki H, Morita I, Suzaki H, Hashimoto M, Sakai T (2008). Comparison of the health condition between the 8020 achievers and the 8020 non-achievers. Int Dent J.

[10] Okamoto Y, Tsuboi S, Suzuki S, Nakagaki H, Ogura Y, Maeda K (2006). Effects of smoking and drinking habits on the incidence of periodontal disease and tooth loss among Japanese males: a 4-yr longitudinal study. J Periodontal Res.

[11] Krall EA, Dawson-Hughes B, Garvey AJ, Garcia RI (1997). Smoking, smoking cessation, and tooth loss. J Dent Res.

[12] Hanioka T, Ojima M, Tanaka K, Aoyama H (2007). Association of total tooth loss with smoking, drinking alcohol and nutrition in elderly Japanese: analysis of national database. Gerodontology.

[13] Albandar JM, Streckfus CF, Adesanya MR, Winn DM (2000). Cigar, pipe, and cigarette smoking as risk factors for periodontal disease and tooth loss. J Periodontol.

[14] Savoca MR, Arcury TA, Leng X, Chen H, Bell RA, Anderson AM (2010). Severe tooth loss in older adults as a key indicator of compromised dietary quality. Public Health Nutr.

[15] Kim S, Park S, Lin M (2017). Permanent tooth loss and sugar-sweetened beverage intake in US young adults. J Public Health Dent.

[16] Tezal M, Grossi SG, Ho AW, Genco RJ (2001). The effect of alcohol consumption on periodontal disease. J Periodontol.

[17] Nakayama Y, Mori M (2012). The relationship between number of natural teeth and oral health behavior in adult Japanese people. J Natl Inst Public Health.

[18] Ishikawa S, Konta T, Susa S, Kitabatake K, Ishizawa K, Togashi H (2019). Risk factors for tooth loss in community-dwelling Japanese aged 40 years and older: the Yamagata (Takahata) study. Clin Oral Investig.

[19] Gaur S, Agnihotri R (2014). Green tea: A novel functional food for the oral health of older adults. Geriatr Gerontol Int.

[20] Koyama Y, Kuriyama S, Aida J, Sone T, Nakaya N, Ohmori-Matsuda K (2010). Association between green tea consumption and tooth loss: cross-sectional results from the Ohsaki Cohort 2006 Study. Prev Med.

[21] Thaper S, Lakshmi T (2017). Effects of mushroom on dental caries. JAPER.

[22] Brennan DS, Singh KA, Liu P, Spencer AJ (2010). Fruit and vegetable consumption among older adults by tooth loss and socio-economic status. Aust Dent J.

[23] Johansson I, Holgerson PL. Milk and oral health. In: Milk and Milk Products in Human Nutrition. Marrakech, Morocco: Karger Publishers; 2011;55–66. 10.1159/000325575 21335990

[24] Krall EA, Wehler C, Garcia RI, Harris SS, Dawson-Hughes B (2001). Calcium and vitamin D supplements reduce tooth loss in the elderly. Am J Med.

[25] Östberg A-L, Nyholm M, Gullberg B, Råstam L, Lindblad U (2009). Tooth loss and obesity in a defined Swedish population. Scand J Public Health.

[26] Nascimento GG, Leite FRM, Conceição DA, Ferrúa CP, Singh A, Demarco FF (2016). Is there a relationship between obesity and tooth loss and edentulism? A systematic review and meta-analysis. Obes Rev.

[27] Al-Shammari KF, Al-Khabbaz AK, Al-Ansari JM, Neiva R, Wang H-L (2005). Risk indicators for tooth loss due to periodontal disease. J Periodontol.

[28] Taguchi A, Sanada M, Suei Y, Ohtsuka M, Lee K, Tanimoto K (2004). Tooth loss is associated with an increased risk of hypertension in postmenopausal women. Hypertension.

[29] Buchwald S, Kocher T, Biffar R, Harb A, Holtfreter B, Meisel P (2013). Tooth loss and periodontitis by socio-economic status and inflammation in a longitudinal population-based study. J Clin Periodontol.

[30] Musacchio E, Perissinotto E, Binotto P, Sartori L, Silva-Netto F, Zambon S (2007). Tooth loss in the elderly and its association with nutritional status, socio-economic and lifestyle factors. Acta Odontol Scand.

[31] Han D-H, Khang Y-H (2017). Lifecourse socioeconomic position indicators and tooth loss in Korean adults. Community Dent Oral Epidemiol.

[32] Nakahori N, Sekine M, Yamada M, Tatsuse T, Kido H, Suzuki M (2019). Socioeconomic status and remaining teeth in Japan: results from the Toyama dementia survey. BMC Public Health.

[33] Center for Healthy Aging and Development Studies. The Chinese Longitudinal Healthy Longevity Survey (CLHLS)-Longitudinal Data (1998–2018). Peking University Open Research Data Platform; 2020.

[34] Zheng Z (2020). Twenty years’ follow-up on elder people’s health and quality of life. China Popul Dev Stud.

[35] Zeng Y. Introduction to the Chinese Longitudinal Healthy Longevity Survey (CLHLS). In: Healthy Longevity in China. Springer; 2008;23–38.

[36] WHO. Obesity and overweight. World Health Organization. 2020. https://www.who.int/news-room/fact-sheets/detail/obesity-and-overweight. Accessed 8 Jul 2021.

[37] Katz S, Ford AB, Moskowitz RW, Jackson BA, Jaffe MW (1963). Studies of illness in the aged: the index of ADL: a standardized measure of biological and psychosocial function. JAMA.

[38] Urzua I, Mendoza C, Arteaga O, Rodríguez G, Cabello R, Faleiros S, et al. Dental caries prevalence and tooth loss in chilean adult population: first national dental examination survey. Int J Dent. 2012;2012. 10.1155/2012/810170 PMC3536045 23316234

[39] Mundt T, Polzer I, Samietz S, Grabe HJ, Dören M, Schwarz S (2011). Gender-dependent associations between socioeconomic status and tooth loss in working age people in the Study of Health in Pomerania (SHIP). Germany Community Dent Oral Epidemiol.

[40] Kim H-N, Ha T-G, Kim M-J, Jun E-J, Jeong S-H, Kim J-B (2016). Factors related to number of present teeth in Korean elderly adults aged 55–84 years. Int J Dent Hyg.

[41] Locker D, Ford J, Leake JL (1996). Incidence of and risk factors for tooth loss in a population of older Canadians. J Dent Res.

[42] Broadbent JM, Thomson WM, Boyens JV, Poulton R (2011). Dental plaque and oral health during the first 32 years of life. J Am Dent Assoc.

[43] Davies RM, Davies GM, Ellwood RP, Kay EJ (2003). Prevention Part 4 Toothbrushing: what advice should be given to patients?. Br Dent J.

[44] Gupta P, Gupta N, Pawar AP, Birajdar SS, Natt AS, Singh HP. Role of sugar and sugar substitutes in dental caries: a review. Int Sch Res Not. 2013;2013. 10.1155/2013/519421 PMC3893787 24490079

[45] Wakai K, Naito M, Naito T, Kojima M, Nakagaki H, Umemura O (2010). Tooth loss and intakes of nutrients and foods: a nationwide survey of Japanese dentists. Community Dent Oral Epidemiol.

[46] Sheiham A (1983). Sugars and dental decay. Lancet.

[47] Burt BA, Ismail AI, Morrison EC, Beltran ED (1990). Risk factors for tooth loss over a 28-year period. J Dent Res.

[48] Jansson L (2008). Association between alcohol consumption and dental health. J Clin Periodontol.

[49] Ando A, Ohsawa M, Yaegashi Y, Sakata K, Tanno K, Onoda T (2013). Factors related to tooth loss among community-dwelling middle-aged and elderly Japanese men. J Epidemiol.

[50] Nakagiri A, Fukushima K, Kato S, Takeuchi K (2006). Less irritative action of wine and Japanese sake in rat stomachs: a comparative study with ethanol. Dig Dis Sci.

[51] Zheng Z, Zhou Y (2014). Health behavior and oral health among elderly people in China. China Popul Dev Stud.

[52] Huang Y, Fan C, Shi Y (2018). Research on prevalence rate and related factors of hypertension and diabetes in senile people based on cross-sectional data of CLHLS study in. J Clin Med Pr.

